# What Is a Colloid?

**Published:** 1921-04-30

**Authors:** 


### What is a Colloid?

### Lecture at the Public Pharmacists' Association.

At a recent meeting of the Public Pharmacists' Association (Mr. Geo. W. Gibson presiding) Mr. J. F. Ward, B.Sc. (Lond.), gave an interesting illustrated lecture on "Colloids in Medicine and Pharmacy." The lecturer discussed the definition of a colloid, and pointed out that the classification, crystalloids and colloids, is a purely arbitrary one. Many substances such as albumen, hæmaglobin, etc., are colloids which can be obtained commercially in crystalline form. Typical crystalloids, as lead iodide, sodium chloride, may be made to assume the colloidal form, whilst a substance like mastich forms a true solution in alcohol, but if this solution is poured into water it assumes the colloidal form. A colloid system must consist of two "phases"—the external or dispersion medium, and the internal or disperse phase. An essential is the insolubility of one in the other. Graham's plan was to designate substances of the class colloids (Gr. *kolla*, glue), and to speak of the peculiar form of aggregative as the "Colloidal Condition of Matter." He affirmed that the colloidal is in fact a dynamical state of matter, the crystalloidal being the statical condition. Colloids possess "energia," and may be regarded as the probable primary source of the force appearing in the phenomena of vitality. Much of our knowledge of colloids is due to the ultra microscope, which enables actual observation of the particles, or rather the reflection, to be made. This apparatus was illustrated and explained, as was the "Brownian Movement," named after Brown the botanist, who observed particles in microscopic preparations to be in a continuous state of rapid oscillatory motion. At the close of the lecture the audience spent an interesting half-hour in viewing the apparatus at close quarters.

